# Data showing levels of interleukin-1β and nitric oxide in the plasma of uropathogenic *E. coli* infected UTI patients

**DOI:** 10.1016/j.dib.2018.05.058

**Published:** 2018-05-18

**Authors:** Vivek Verma, Renu Arora, Rakesh Singh Dhanda, Rajni Gaind, Manisha Yadav

**Affiliations:** a Dr. B. R. Ambedkar Center for Biomedical Research (ACBR), University of Delhi, Delhi 110007, India; b Department of Obstetrics and Gynecology, Vardhman Mahavir Medical College (VMMC) and Safdarjung Hospital, Delhi 110029, India; c Stem Cell Laboratory, Longboat Explorers AB, SMiLE Incubator, Scheelevägen 2, 22381 Lund, Sweden; d Department of Microbiology, Vardhman Mahavir Medical College (VMMC) and Safdarjung Hospital, Delhi 110029, India

**Keywords:** Uropathogenic *Escherichia coli*, Interleukin-1β, Nitric oxide

## Abstract

Urinary tract infections (UTI) are a major cause of morbidity, affecting at least four million women worldwide, 65–75% of these infections are caused by Uropathogenic *Escherichia coli* (UPEC) (Foxman, 2010) [1]. Repertoire of virulence factors carried by UPEC provides the ability to precede urinary tract and additionally they provoke pro-inflammatory responses (Cirl et al., 2008; Verma et al., 2016) [2], [3]. In context to UPEC infected UTI patients, the levels of pro-inflammatory cytokine IL-1β and enzymatic antioxidant nitric oxide (NO) have not been reported worldwide till date, including India. In this data article, we report for the first time the levels of IL-1β and nitric oxide in the plasma of UPEC infected UTI patients. Data includes a profile of pro-inflammatory cytokine IL-1β and NO in the plasma of the confirmed UPEC infected UTI patients (*N* = 30) versus healthy controls (*N* = 40) from the present pilot study. The levels of IL-1β in plasma were significantly higher (*p* < 0.0001) in patients (252.3 ± 6.49 pg/ml) as compared to healthy controls (127.6 ± 3.98 pg/ml), whereas plasma levels of NO were significantly lower (*p* < 0.0001) in UPEC infected UTI patients (60.29 ± 1.1 μM) as compared to healthy controls (106.3 ± 8.75 μM).

## Specifications Table

**Table:** 

Subject area	*Biology*
More specific subject area	*Medical Microbiology, Uropathogenic Escherichia coli*
Type of data	*Graphs*
How data was acquired	*ELISA and Griess reagent assay*
Data format	*Analyzed*
Experimental factors	*Confirmed UPEC infected UTI patients*
Experimental features	*IL-1β and NO levels in UPEC infected UTI patients*
Data source location	*New Delhi, India*
Data accessibility	*Data is with this article only*

## Value of the data

• First report of cytokine IL-1β and NO levels in plasma of UPEC infected UTI patients in the Indian population.
• Data shows significant increase in the levels of pro-inflammatory cytokine IL-1β and decreased antioxidant NO levels in plasma of UPEC infected UTI patients as compared to healthy controls.
• An elevated level of IL-1β in patients may indicate the induction of pro-inflammatory pathway in host to resolve infection through recruitment of polymorphonuclear cells.
• Decreased levels of NO in UPEC infected UTI patients may indicate the presence of higher levels of free radicals in circulating blood plasma.

## 1 Data

**Fig. 1:**
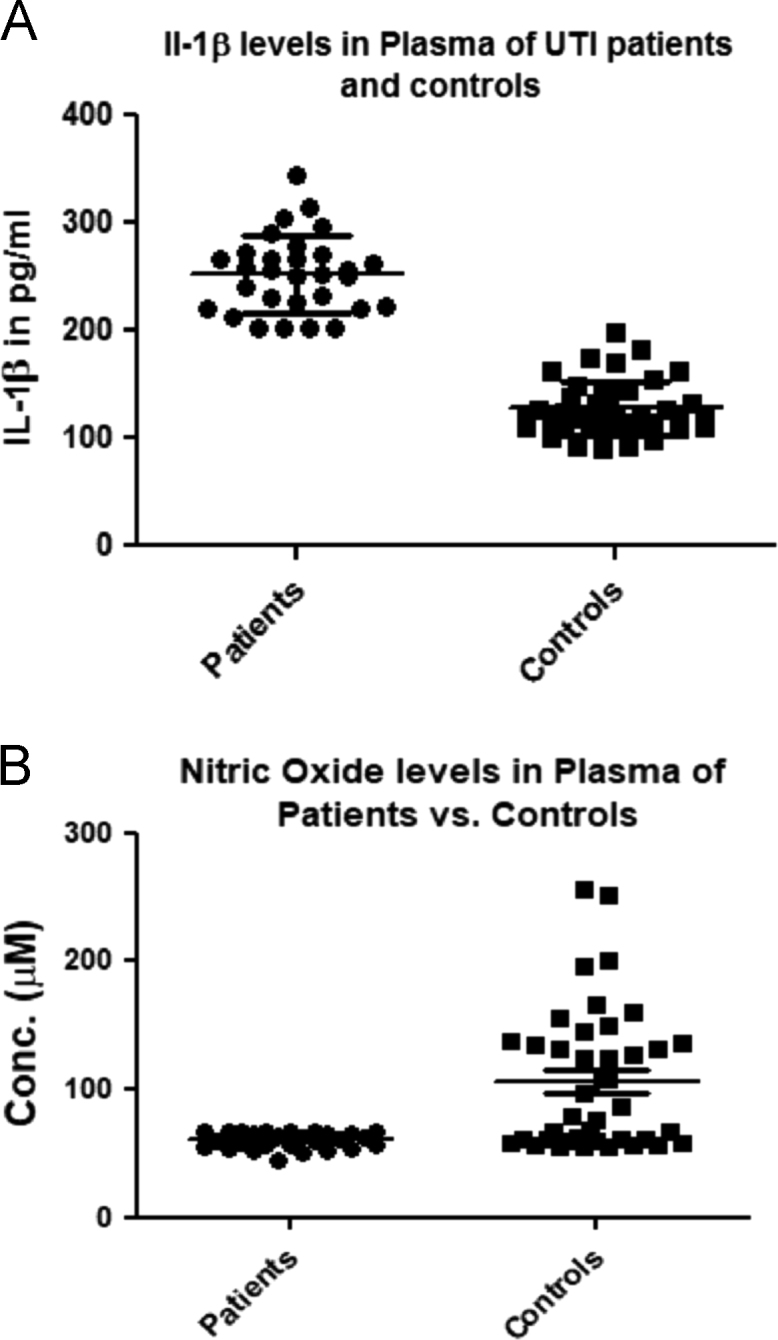
(A) Graph represents the grouped scattered plot of Plasma levels of pro-inflammatory cytokine IL-1β (pg/ml) in the UTI patient versus Control group. *P* value < 0.0001; (B) Graph represents the grouped scattered plot of NO levels (μM) in the UTI patient versus Control group. *P* value < 0.0001.

The distribution of IL-1β and NO levels in plasma from individual subjects is shown in Fig. 1A and B respectively as grouped scatter plot, data is shown as Mean±SEM. Plasma level of pro-inflammatory cytokine IL-1β in UPEC infected UTI patients (252.3 ± 6.49 pg/ml) is significantly higher than that of healthy control groups (127.6 ± 3.98 pg/ml) (*P *< 0.0001). IL-1β level was found to be 2 times higher in patients as compared to that of controls (Fig. 1A). NO levels in plasma of UPEC infected UTI patients (*N* = 30) was significantly lower (60.29 ± 1.1 μM) as compared to healthy controls (106.3 ± 8.75 μM). Mean value of concentration of NO levels were 1.7 times lower in patients as compared to the controls group (*P* < 0.0001) (Fig. 1B).

## 2 Experimental design, materials, and methods

### 2.1 Study population and sample collection

A total of 70 study subjects were included for the present study. Total of 30 women patients were recruited from the outpatient department (OPD) at Department of Obstetrics and Gynecology, Vardhman Mahavir Medical College (VMMC) & Safdarjung Hospital, New Delhi, India. The patients attending OPD with the complaints of burning micturition, frequent dysuria, abdominal pain, loin tenderness, dysfunctional voiding, hematuria and fever were considered as UTI patients. All the patients were screened for UPEC infection through Dip Stick test, biochemical analysis, microscopy and routine urine culture by examining their urine in the Department of Microbiology, VMMC & Safdarjung Hospital, New Delhi, India. Among these patients, only those fulfilling following criteria were enrolled in the study: 1) patients aged 18–60 years, [2] first episode of UTI and [3] positive urine culture ≥ 10^5^ CFU of UPEC (single micro-organism). From culture positive patients, 5 ml of blood was drawn and their urine samples were taken for culture/microscopic studies. Patients suffering from metabolic diseases (hypertension, arthritis, diabetes, Hypo/hyper thyroidism etc.) and any other simultaneous infections other than UPEC caused UTI were excluded from the study. Patients having problem of urolithiasis or antibiotic use in the prior week and pregnant women were also not included. Patients which have undergone any surgical procedure or recent catheterization, or known anatomic or functional abnormalities of the urinary tract were also excluded.

The control group consisted of 40 females (aged between 25 and 60 years) who visited the OPD for routine checkup or family planning advices were included as healthy controls. Volunteers having any infection or metabolic diseases were excluded. Written informed consent was obtained from all the participants recruited for this study. An approval from institutional ethics committee of VMMC & Safdarjung Hospital, New Delhi, India (No. IEC/SJH/VMMC/Project/Sept-14/490) and Dr. B. R. Ambedkar Center for Biomedical Research, University of Delhi (No. F50-2/Eth. Com/ACBR/15) were obtained prior to the study.

### 2.2 Cytokine measurement

Five milliliter (5 ml) of peripheral venous blood was collected from all the participants in an ethylene diamine-tetra acetic acid (EDTA) vial. Separation of plasma was done by centrifugation at 520 *g* for 10 min at 4 °C. Plasma samples were stored in − 80 °C for further use. Cytokine IL-1β was quantified by enzyme-linked immunosorbent assay (ELISA) using Ready-SET-Go ELISA kits (eBiosciences, San Diego, USA) as per the manufacturer׳s instructions.

### 2.3 Estimation of NO levels

NO level was estimated in the plasma samples of both UTI patients and control group using Griess reagent method [4].

## 3 Statistical analysis

All statistical analysis was performed using GraphPad Prism version 5 (GraphPad Software Inc., San Diego, CA, USA). Patients group was compared with healthy controls using unpaired student *t*-test. Results were expressed as Mean ± SEM. *P*-value < 0.05 was considered as statistically significant.
